# Memory and Energy Optimization Strategies for Multithreaded Operating System on the Resource-Constrained Wireless Sensor Node

**DOI:** 10.3390/s150100022

**Published:** 2014-12-23

**Authors:** Xing Liu, Kun Mean Hou, Christophe de Vaulx, Jun Xu, Jianfeng Yang, Haiying Zhou, Hongling Shi, Peng Zhou

**Affiliations:** 1 LIMOS Laboratory, CNRS UMR 6158, Blaise Pascal University, Les Cézeaux, BP 10125, Clermont-Ferrand 63173, France; E-Mails: liu@isima.fr (x.L.); devaulx@isima.fr (C.V.); shi@isima.fr (H.S.); peng.zhou@isima.fr (P.Z.); 2 Internet and Information Technology Laboratory, Electronic Information School, Wuhan University, Road LuoJia, Wuhan 430072, China; E-Mail: eisxujun@whu.edu.cn; 3 School of Electrical & Information, Hubei University of Automotive Technology, Shiyan 442002, China; E-Mail: haiyingzhou@hit.edu.cn

**Keywords:** memory optimization, energy conservation, operating system, wireless sensor network, multi-core

## Abstract

Memory and energy optimization strategies are essential for the resource-constrained wireless sensor network (WSN) nodes. In this article, a new memory-optimized and energy-optimized multithreaded WSN operating system (OS) LiveOS is designed and implemented. Memory cost of LiveOS is optimized by using the stack-shifting hybrid scheduling approach. Different from the traditional multithreaded OS in which thread stacks are allocated statically by the pre-reservation, thread stacks in LiveOS are allocated dynamically by using the stack-shifting technique. As a result, memory waste problems caused by the static pre-reservation can be avoided. In addition to the stack-shifting dynamic allocation approach, the hybrid scheduling mechanism which can decrease both the thread scheduling overhead and the thread stack number is also implemented in LiveOS. With these mechanisms, the stack memory cost of LiveOS can be reduced more than 50% if compared to that of a traditional multithreaded OS. Not is memory cost optimized, but also the energy cost is optimized in LiveOS, and this is achieved by using the multi-core “context aware” and multi-core “power-off/wakeup” energy conservation approaches. By using these approaches, energy cost of LiveOS can be reduced more than 30% when compared to the single-core WSN system. Memory and energy optimization strategies in LiveOS not only prolong the lifetime of WSN nodes, but also make the multithreaded OS feasible to run on the memory-constrained WSN nodes.

## 1. Introduction

A wireless sensor network (WSN) consists of distributed wireless sensor nodes which monitor the environmental conditions (temperature, sound, pressure, *etc.*) and send the collected data cooperatively through the network to a main location (e.g., the sink node) [1,2]. Currently, WSN technology has played a significant role in daily life [3,4] and is viewed as one of the key technologies of the 21st century [5].

For widespread use in different application domains, WSN nodes need to be small and inexpensive. Consequently, WSN nodes are mostly constrained in the memory resource (e.g., MicaZ node has only 4 KB RAM) and energy resource (most nodes are powered by small-sized batteries) [2]. Therefore, the memory and energy optimization strategy becomes essential for the WSN node. With these optimizations, the lifetime of the WSN nodes can be prolonged. Moreover, a software system, which has high memory cost, can become more feasible to run on the nodes.

**Figure 1.**
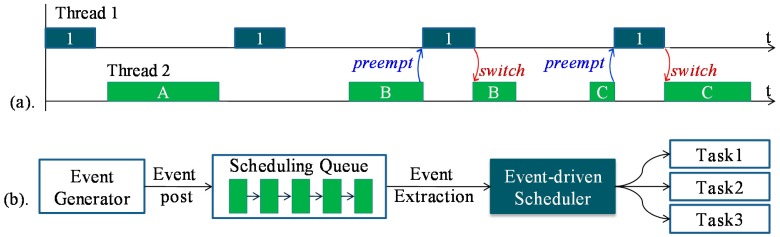
(**a**) Multithreaded scheduling system. Threads can preempt each other, thus each thread needs to have an independent stack; (**b**) Event-driven scheduling system. Events which are triggered will be posted into the scheduling queue, and then be withdrawn one by one by the scheduler. Tasks cannot preempt each other, and only one shared run-time stack is needed.

An operating system (OS) is important for the WSN as it can manage the hardware resources and serve the application development. The current WSN OSes can be classified into two kinds: event-driven OS and multithreaded OS [6,7]. In the event-driven OS, preemption is not supported; one task can be executed only after the previous one runs to completion (Figure 1b). Due to this feature, memory cost of event-driven OS is low [8]. However, tasks in the event-driven OS cannot be blocked during the run-time, thus split-phase state-machine programming is commonly needed [9], and this is difficult for common users to manipulate. Moreover, the real-time performance of the event-driven system is poor, since the time-critical task cannot be executed immediately by the preemption. Multithreaded OS is different from the event-driven OS in that several tasks can run concurrently, and the split-phase programming is not necessary. Moreover, real-time scheduling can be achieved by using the thread preemption (Figure 1a). Since the preemption can be performed, each task in the multithreaded OS needs to have an independent run-time stack. Consequently, memory cost of the OS becomes high, and this makes the multithreaded OS not suitable to run on the severe resource-constrained WSN nodes. Therefore, the memory optimization mechanism becomes significant for the multithreaded OS, especially on the memory-constrained WSN nodes. Besides the memory optimization, energy optimization is also critical for a multithreaded WSN OS [2]. With the energy optimization, the lifetime of WSN nodes can be prolonged. As a result, the work of recollecting all the deployed nodes at intervals to recharge the energy can be eased. This is quite essential for the nodes deployed outdoors in harsh environments.

In this article, a new multithreaded WSN OS LiveOS (former name MIROS) is designed and implemented. LiveOS aims to be a both memory-efficient and energy-efficient multithreaded WSN OS. To achieve the memory-efficient objective, LiveOS uses the stack-shifting hybrid scheduling mechanism. In contrast to a traditional multithreaded OS in which the stacks are allocated statically by the pre-reservation, stacks in LiveOS are allocated dynamically in terms of the run-time requirement. By doing this, memory waste caused by the static pre-reservation can be avoided. Since the dynamic-stack scheduling has higher run-time overhead, the hybrid scheduling mechanism, which can reduce the thread preemption frequency, is implemented in LiveOS. With the hybrid scheduling, the memory cost of the dynamic-stack scheduling can be decreased, while the performance of the dynamic-stack scheduling is maintained. To achieve the energy-efficient objective, LiveOS implements two energy-conservation approaches: the multi-core “context-aware” approach and the multi-core “power-off/wakeup” approach. As opposed to the other conservation approaches in which energy is conserved only from the software aspect, energy conservation in LiveOS is achieved from both the software aspect and the multi-core hardware aspect. By so doing, energy utilization efficiency is improved and the lifetime of WSN nodes can be prolonged.

The structure of this paper is organized into five sections: In Section 2, the related works about the memory and energy optimization approaches are introduced. In Section 3, the LiveOS stack-shifting hybrid scheduling mechanism is investigated and evaluated. In Section 4, two LiveOS multi-core energy conservation approaches are presented and evaluated. Finally in Section 5, a conclusion is made and ongoing work is proposed.

## 2. Related Works

### 2.1. Memory Optimization of the Thread Stacks in Multithreaded Wireless Sensor Network Operating Systems

In this section, two kinds of stack memory optimization approaches are presented. One is the stack-size analysis approach [10,11] (Section 2.1.1). This approach is currently being used in the traditional multithreaded WSN OS mantisOS [12]. The other is the cooperative multithreading approach (Section 2.1.2). This approach is currently being used in several multithreaded Java OSes.

#### 2.1.1. Stack-Size Analysis Approach

**Figure 2.**
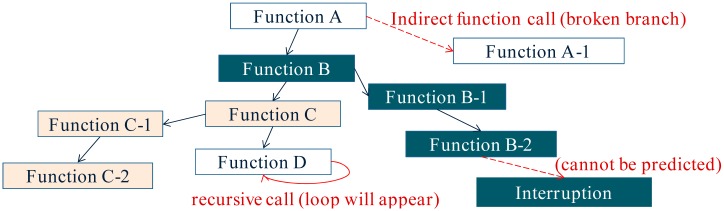
The thread control-flow graph modeled by the stack-size analysis approach. Stack usage can be calculated by the straightforward depth-first search in this graph.

In a traditional multithreaded OS, the size of each stack is pre-reserved heuristically. If the reserved size is too small, memory overflow will occur. To avoid this problem, stack size is commonly reserved to a large value, e.g., 128 bytes in the mantisOS (on the 8-bit AVR microcontroller). However, memory waste will occur in this case. Ideally, the stack size will be reserved to the minimal but system-safe size, and this is the objective of the stack-size analysis approach. With the stack-size analysis approach, the thread execution process can be modeled as a control-flow graph. The thread functions and the local stack usages represent the nodes in the graph, whereas the branch instructions represent the edges in the graph (Figure 2). After this model is built, the stack usage of each thread can be calculated by the straightforward depth-first search. During the search process, if a “PUSH” or “CALL” instruction is observed, the stack usage value will increase. Otherwise, if a “POP” instruction is met, the stack usage value will decrease. By doing this, the stack size which will be required during the run-time can be computed, and the stack memory waste caused by the heuristic pre-reservation approach can be avoided.

With the stack-size analysis approach, the thread memory cost can be optimized. However, there are several cases in which the stack usage cannot be calculated, such as interruption reentrancy, recursive calls and indirect function calls. If interrupt is reenterable, the number of received interruptions during the run-time cannot be pre-known. If recursive calls exist, the cycle will appear in the control-flow graph. If indirect function calls exist, the disconnection will appear in the control-flow graph (Figure 2). To solve these problems, several solutions are proposed in the article [10]. However, the final evaluation results show that these solutions are not ideal, although the guesswork in determining the stack size is eliminated. As a result, stack-size analysis approach is not effective in some WSN systems.

#### 2.1.2. Cooperative Multithreading Approach

The cooperative multithreading approach is commonly used to solve the deadlock and synchronization problem in the multithreaded OS. However, it can also be used to optimize the memory cost of thread stacks. Typically, this optimization approach has been used in the Embedded Java Virtual Machine (EJVM). EJVM is attractive to the WSN as it not only simplifies the WSN programming process (programming by using Java without considering the low-level details), but also improves the WSN reprogramming performance (only Java application image rather than the monolithic software image needs to be updated during the reprogramming process). Currently, the cooperative multithreading approach (also named “atomic instruction” or “bytecode-granularity switch” in the EJVM terminologies) has been applied in several EJVMs, including the simpleRTJ [13], the Darjeeling VM [14] and the TakaTuka JVM [15].

**Figure 3.**
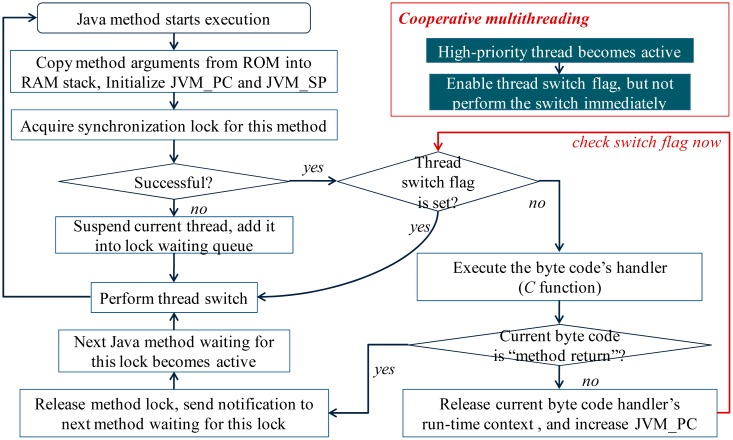
Elementary diagram of the EJVM simpleRTJ. Java thread switch is performed cooperatively in simpleRTJ. Only after the byte code's handler runs to completion and releases its run-time context, can the Java thread switch be performed.

**Figure 4.**
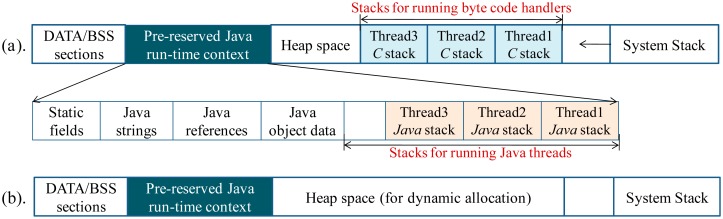
(**a**) If the Java thread can preempt each other at any time, each C-programmed byte code handler needs to have an independent run-time stack; (**b**) After the cooperative threading mechanism is used, byte code handlers will not be preempted during the mid-executing process. Consequently, no independent C run-time stack needs to be pre-reserved for each thread.

The elementary diagram of EJVM simpleRTJ is depicted in the Figure 3. After the Java method is initialized, the method byte code will be interpreted one by one. Each byte code has a corresponding bytecode handler (programmed in *C* language). The byte code handler will be invoked once a Java byte code is interpreted. In simpleRTJ, the thread switch is performed in a cooperative way. After a high-priority Java thread becomes active, the preemption will not be performed immediately. Instead, only the thread switch flag is set. Once the byte code handler runs to completion, the thread switch flag will be checked to be set or not. If no, the next byte code will be interpreted continuously. If yes, the thread switch will be performed. Since thread switch will not be performed during the mid-executing process of the *C*-programmed byte code handlers, the run-time contexts of these handlers need not be saved when the thread switch is performed. Consequently, memory cost of the multithreaded system can be reduced significantly (Figure 4).

With the cooperative multithreading approach, the memory consumption of the multithreaded OS can be optimized. However, the preemption cannot be performed immediately, thus the real-time performance of the OS decreases. Due to this reason, this approach is not suitable to optimize the memory cost in the real-time WSN applications (industrial engine control, smart care, contaminant detection, *etc.*).

### 2.2. Energy Conservation to the WSN Node

The energy conservation strategy is significant for the WSN nodes for two key reasons. First, many WSN nodes are deployed outdoors and need to be powered by small-sized batteries; the available energy resource is therefore limited. Second, WSN nodes are prone to be deployed in the harsh environment where human access is difficult; thus the recollection of the deployed nodes to recharge the energy is quite difficult. Since the lifetime of the nodes is determined by the energy supply, the energy conservation mechanism is essential to the WSN nodes. Currently, the “sleep/wakeup” approach is used popularly in most WSN OSes to conserve energy resources, and this approach has been achieved through two aspects: the OS scheduling aspect and the network protocol aspect. In TinyOS [16], Contiki [17] and SOS [18], the “sleep/wakeup” mechanism is achieved from the scheduling aspect. A scheduling queue is polled in these OSes by the event-driven scheduler. In the case that no events are pending in the queue, the sleep directive will be called, and then the node will fall asleep to conserve energy. In openWSN [19], the “sleep/wakeup” mechanism is also applied, but it is achieved from the network protocol aspect. In openWSN, the IEEE 802.15.4e protocol is developed [20]. With this protocol, the WSN nodes can wake up synchronously to communicate with each other within a given period, whereas fall asleep or power off the transceivers during the other periods.

The “sleep/wakeup” approach can conserve the node energy resource in a degree. However, the conservation effect is not adequate. Currently, the energy constraint is still a critical challenge for the WSN nodes [2], and the research of the new conservation approaches is still significant.

## 3. LiveOS Memory Optimization by the Dynamic-Stack Hybrid Scheduling Mechanism

In Section 2, the stack-size analysis approach and cooperative multithreading approach are introduced. Although these approaches can reduce the memory cost of multithreaded OSes, they are either unavailable in many cases (stack-size analysis approach), or will decrease significantly the OS real-time performance (cooperative multithreading approach). In this section, the LiveOS memory optimization strategy, which is achieved by using the stack-shifting hybrid scheduling mechanism, is investigated. In Sections 3.1 and 3.2, the concept and implementation of the LiveOS dynamic-stack scheduling are presented respectively. In Sections 3.3 and 3.4, the performance improvement work and the evaluation work to the LiveOS dynamic-stack scheduling are presented respectively. In Section 3.5, a discussion on the different memory optimization approaches is presented.

### 3.1. Concept of LiveOS Dynamic-Stack Scheduling

**Figure 5.**
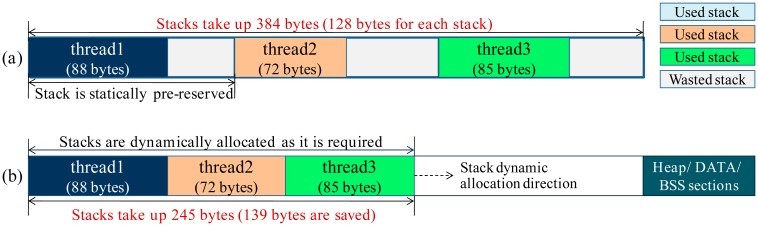
(**a**) In traditional multithreaded OS, stacks are statically pre-reserved, and the stack size is assigned heuristically. Memory waste should exist in this case; (**b**) In LiveOS, stacks are allocated dynamically within a shared stack. By doing this, memory waste can be avoided. In this example, 139-byte memory is saved by LiveOS.

In traditional multithreaded OSes, each thread runs in a statically pre-reserved stack. If the reserved stack size is small, the stack will overflow during the run-time. To avoid this problem, stack size needs to be assigned to a large value which can meet the requirement of the worst case (stack size is commonly assigned by heuristically). However, memory waste will be caused in this way, e.g., in Figure 5a, each thread stack is statically assigned to 128 bytes, but some stack memory will never be used during the run-time. Stack-size analysis attempts to avoid the memory waste by assigning the stack size to the minimal but system-safe value, however, this approach is not available in some cases (Section 2.1.1). In LiveOS, stacks are designed to be dynamically allocated so that the memory waste problem can be avoided. Different from the traditional multithreaded OS in which each thread runs in its pre-reserved independent stack, in LiveOS all the threads run in a shared stack. As illustrated in Figure 5b, if thread2 preempts thread1, the stack switch will not be performed (In traditional multithreaded OS, stack pointer will switch to the thread2's stack area in this case). Instead, thread1 saves its context, and then thread2 continues to run in the memory area adjacent to the thread1's stack. If thread3 preempts thread2, the same operation will be performed. By doing this, the memory size of each stack in LiveOS will be equal to the actual size that is required, and the memory waste problem in the static-stack mechanism can be avoided.

### 3.2. Implementation of LiveOS Dynamic-Stack Scheduling by Using the Stack Shifting Technique

**Figure 6.**
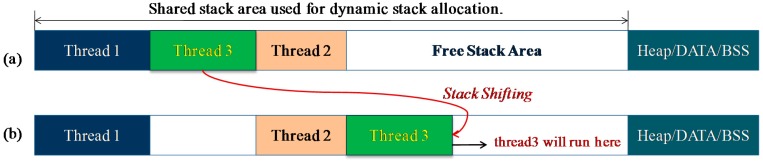
If thread3 needs to be executed, its stack should be shifted so that it will not corrupt the context data of thread2 during the run-time.

After the dynamic-stack approach is used, all the thread stacks are allocated dynamically within a shared stack space, and the stack memory cost can be reduced. However, thread stacks are saved continuously in the shared stack, rather than independently in the separated pre-reserved stack. Thus, the execution of one thread can cause another thread's context to be corrupted. For example, in Figure 6a, no free memory space exists between thread3's stack and thread2's stack, thus the context data of thread2 will be corrupted if thread3 resumes its execution. To solve this problem, the stack-shifting technique is used in LiveOS. With this technique, the stack of the next thread to be scheduled will be shifted to the free stack area. By doing this, threads can be executed safely without the risk of corrupting the others' stacks, e.g., if thread3 needs to be executed in Figure 6a, its stack will be shifted to the free stack area (Figure 6b).

**Figure 7.**
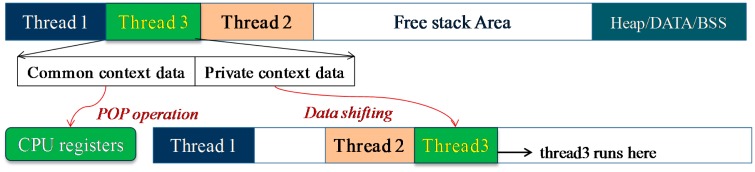
Stack shifting process in LiveOS. Only the private context data rather than the whole context data needs to be shifted during the stack-shifting operation.

In each thread stack, two kinds of context data are included: the private context data and the common context data. Private context data contains the value of the local variables and the CPU registers which have been used. Common context data contains the CPU status register and all the CPU general registers. When the thread switch is performed in the traditional static-stack multithreaded system, the common context data needs to be restored (by the “POP” operation). However, after the LiveOS dynamic-stack scheduling mechanism is used, not only the common context data needs to be restored, but also the private context data needs to be shifted during the thread switch process (Figure 7). Namely, the advantage of LiveOS's lower stack memory cost is achieved at the cost of higher thread switch overhead.

**Figure 8.**
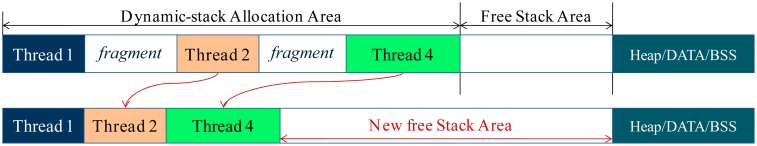
Memory defragmentation in the dynamic-stack allocation area. After the memory fragments are coalesced, the free stack area can become larger.

After the stack-shifting operations are performed for some time, fragmentation can occur in the dynamic-stack allocation area, and the size of the free stack area will decrease (Figure 8). If the size of free stack area decreases to a given value, the stack-shifted thread can no longer run safely. In this case, memory defragmentation to the dynamic-stack allocation area needs to be performed. In LiveOS, the defragmentation is achieved by coalescing the allocated stack memory within the dynamic-stack allocation space (Figure 8). After the defragmentation, free stack area can become larger. Currently, the LiveOS stack memory defragmentation is managed by the LiveOS dynamic allocator (memory defragmentation operation in LiveOS stack space is the same as that in LiveOS heap space). The current LiveOS allocator is improved on the base of the mantisOS sequential fit allocator [21] (by extending the defragmentation functionality). In case no appropriate free item can be found in the free memory list, the defragmentation operation will be performed. With the defragmentation functionality, memory utilization efficiency of LiveOS can be improved and the allocation failure probability decreased.

**Figure 9.**
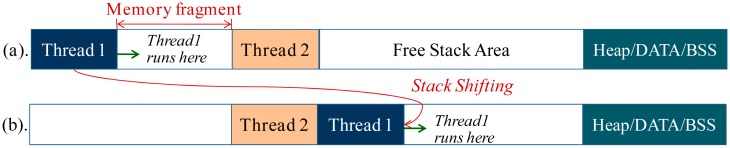
Stack shifting can be avoided if the size of the fragment is large enough for the thread to run. (**a**) Fragment space is large enough, thus stack of thread1 needs not be shifted; (**b**) Stack of thread1 needs to be shifted if fragment space is not large enough.

There exist two typical cases in which the stack shifting needs not to be performed during the thread switch process. One is when the thread scheduling sequence is opposite to the thread stack saved sequence by chance. For example, in Figure 6a, if the thread scheduling sequence is “thread2, thread3, thread1” (assuming that each thread releases its stack after it runs to the completion), the stack shifting will not be needed. The other case is when the memory fragment space adjacent to the thread stack is large enough for this thread to run, e.g., in Figure 9, stack of thread1 needs not be shifted if the fragment space is large enough for it to be executed. Since the stack size required by a thread during its run-time is difficult to pre-calculate in many cases (Section 2.1.1), this approach is available only when the fragment space is quite large and it can be ensured that stack memory will not overflow.

### 3.3. Performance Improvement to the Dynamic-Stack Mechanism by Using Hybrid Scheduling

The stack needs to be shifted when the thread is switched in LiveOS. As a result, the scheduling overhead of LiveOS becomes higher. To improve the LiveOS scheduling performance, the stack-shifting frequency needs to be optimized. Currently, this optimization has been achieved in LiveOS by using the hybrid scheduling mechanism.

**Figure 10.**
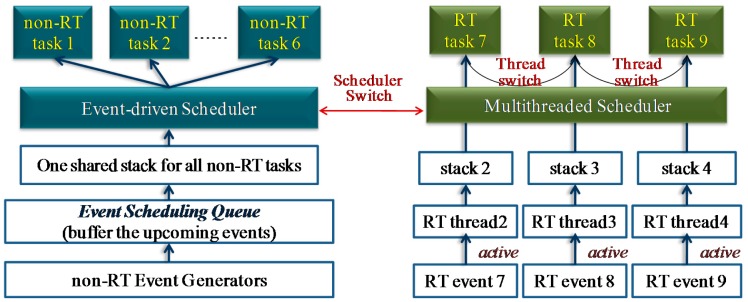
LiveOS hybrid scheduling mechanism. In the traditional pure multithreaded OS, the high-priority non-RT task can preempt the low-priority non-RT task. In LiveOS, the hybrid scheduling mechanism is implemented, and non-RT tasks cannot preempt each other. Non-RT tasks in LiveOS are programmed by using the protothread [22] approach.

The motivation of LiveOS hybrid scheduling derives from the fact that thread preemption is commonly needed among the real-time (RT) threads (a high-priority time-critical thread preempts a low-priority thread so that it can be executed immediately), rather than among the non-RT threads (non-RT threads have loose requirement to the execution response time; they can be executed one by one without preempting each other). Thus, two kinds of schedulers can be implemented in the WSN OS: the multithreaded scheduler and the event-driven scheduler. And the system tasks can be classified into two types: the RT tasks and the non-RT tasks. RT tasks have the preemption requirement, thus they can be scheduled by the multithreaded scheduler. Non-RT tasks have less time constraint, thus they can be scheduled by the event-driven scheduler (Figure 10). With the hybrid scheduling mechanism, preemption will no longer be performed among the non-RT tasks. Consequently, the thread switch frequency is decreased, and then the stack-shifting frequency can be reduced. By doing this, the LiveOS dynamic-stack scheduling performance can be improved.

**Figure 11.**
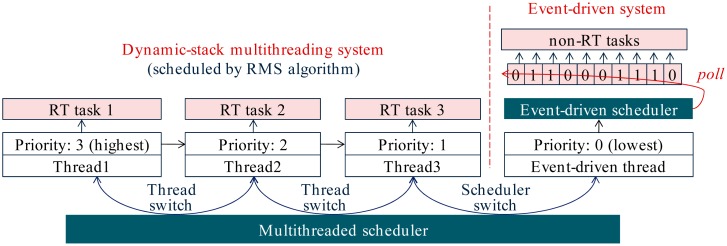
An event-driven scheduling system in LiveOS is also implemented as a thread. Since all non-RT tasks are executed by one thread (event-driven thread), thread number (or stack number) in LiveOS is small. In LiveOS, threads are scheduled by the RMS algorithm. When thread switch is performed, the first ready thread in the queue will be the next one to be executed.

In LiveOS, the Rate-Monotonic Scheduling (RMS) algorithm [23] is used to schedule the RT tasks. RMS is selected because it uses the static scheme, it is simple to implement, and the scheduling efficiency is high. After the hybrid scheduling mechanism is applied, two kinds of scheduling switches exist in LiveOS. One is the thread switch among the RT tasks, the other is the scheduler switch between the event-driven scheduler and the multithread scheduler (Figure 10). In order to perform these two kinds of scheduling switches efficiently, the event-driven system in LiveOS is also implemented as a thread “event-driven thread.” By so doing, the hybrid scheduler can be implemented in the way that the pure multithreaded scheduler is done. As a result, not only the hybrid scheduling implementation complexity will be decreased, but also the hybrid scheduling efficiency will be improved. As illustrated in Figure 11, all non-RT tasks are executed by the event-driven thread. Event-driven thread has the lowest priority, and it can be preempted by any RT thread. If all the RT threads are inactive, the event-driven thread will be scheduled. In this case, LiveOS runs in the event-driven scheduling model, and all non-RT tasks will be executed one by one. However, if any RT thread becomes active, the event-driven thread will be preempted, and then LiveOS switches to the multithreaded scheduling model.

By using the hybrid scheduling mechanism, the performance of LiveOS dynamic-stack scheduling can be improved. It is assumed that five threads (*T1*, *T2*, *T3*, *T4*, *T5*) exist in LiveOS, and these threads have the following features:

(1) *T1* and *T2* are RT threads while the others are non-RT threads.
(2) The thread priority (PRI) is as follows: PRI(*T1*) > PRI(*T2*) >PRI(*T3*) >PRI(*T4*) > PRI(*T5*).
(3) The thread execution process is as follows:

At time t1, *T1* becomes ready and preempts *T2*; at time t2, *T1* suspends and *T5* starts to run; at time t3, *T4* becomes ready and preempts *T5*; at time t4, *T3* becomes ready and preempts *T4*; at time t5, *T2* becomes ready and preempts *T3*; at time t6, *T2* runs to completion and releases its run-time context. Then, *T4* resumes its execution; at time t7, *T1* becomes ready again and preempts *T4*; at time t8, *T1* runs to completion and releases its run-time context. *T3* then resumes its execution; at time t9, *T3* runs to completion and releases its run-time context. *T4* subsequently resumes its execution; at time t10, *T4* runs to completion and releases its run-time context. *T5* then resumes its execution. Finally, *T5* runs to completion and releases its run-time context.

**Figure 12.**
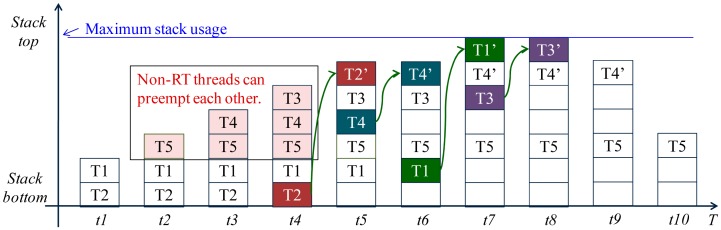
The thread scheduling process and the maximum stack usage in LiveOS before the hybrid scheduling mechanism is implemented.

**Figure 13.**
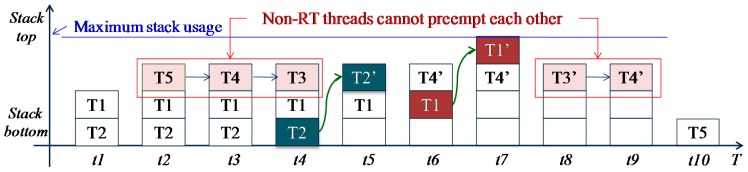
The thread scheduling process and the maximum stack usage in LiveOS after the hybrid scheduling mechanism is implemented. Since non-RT tasks are executed one by one cooperatively, the thread switch frequency and the maximum stack usage are both decreased.

If the above five threads are executed by LiveOS without the hybrid scheduling optimization, the scheduling process will be as that in Figure 12. However, if the hybrid scheduling approach is used for the performance optimization, the thread scheduling process will be as that in Figure 13.

From Figures 12 and 13, it is shown that the stack-shifting counts are reduced by the hybrid scheduling mechanism from four times (t4, t5, t6, t7 in Figure 12) to two times (t4, t6 in Figure 13). The decrease of the stack-shifting frequency will improve the LiveOS dynamic-stack scheduling efficiency.

Besides the reduction of the stack-shifting overhead, the implementation of the hybrid scheduling can also decrease the stack memory cost of LiveOS. This is because all non-RT tasks will be executed by only one thread after the hybrid scheduling mechanism is applied (Figures 10 and 11). By doing this, the stack number in LiveOS can be decreased. With the decreasing of the stack number, the stack memory cost in LiveOS will be significantly reduced. In case the hybrid scheduling is not applied in LiveOS, the maximum stack memory usage of the five threads in Figure 12 is:

(1) $$\text{Stack}\_\text{usage}\_A=(T2+T1)+(T5+T4+T3)+\max(T2^{\prime },T1^{\prime }+T4^{\prime },T3^{\prime }+T4^{\prime })$$

However, after the hybrid scheduling is implemented, the maximum stack memory usage of the five threads in Figure 13 will be:

(2) $$\text{Stack}\_\text{usage}\_A^{\prime }=(T2+T1)+\max\left(T5,T4,T3\right)+\max\left(T2^{\prime },T1^{\prime }+T4^{\prime }\right)$$

Since *Stack*_*usage*_*A′* is smaller than *Stack*_*usage*_*A*, stack memory cost is decreased by using the hybrid scheduling mechanism.

From the evaluation above, it is shown that the hybrid scheduling not only optimizes the LiveOS dynamic-stack scheduling overhead, but also optimizes the LiveOS stack memory cost. With the combination of the dynamic-stack scheduling and the hybrid scheduling, LiveOS becomes more feasible to be used on the resource-constrained WSN nodes.

### 3.4. Performance Evaluation

**Figure 14.**
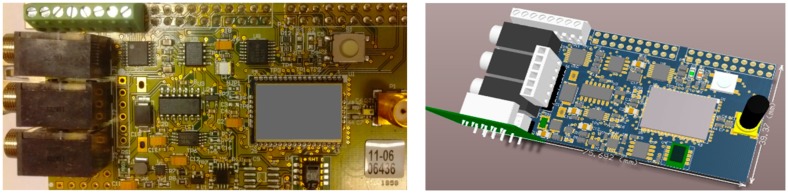
Prototype board of iLive node. iLive is equipped with the ATmega1281 microcontroller as well as 11 sensors (one temperature sensor, one light sensor, one air sensor, one humidity sensor, three soil moisture decagon sensors [26] and four soil moisture watermark sensors [27]).

In this section, the scheduling performance of LiveOS is evaluated by comparing with that of the other WSN OSes from the perspectives of code memory cost, data memory cost and scheduling efficiency. The evaluation is performed on the iLive node (Figure 14) and the AVR studio [24] is used as the software development tool.

#### 3.4.1. Code Memory Size of the Multithreaded Scheduling

**Figure 15.**
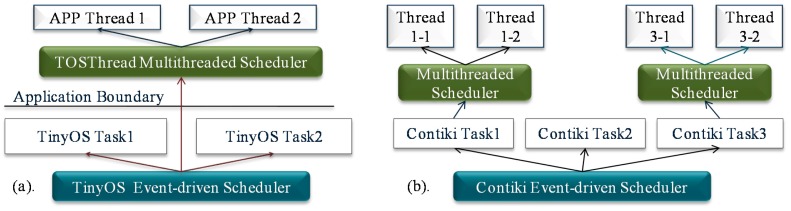
Multithreaded scheduling system in TinyOS (**a**) and Contiki (**b**). Different from LiveOS in which the event-driven scheduler and the multithreaded scheduler are implemented in parallel, the multithreaded scheduler in TinyOS and Contiki is implemented on the top of the event-driven scheduler. Since the event-driven scheduler is still used in the native scheduling layer, the real-time scheduling cannot be achieved in these two OSes as of yet.

The current popular WSN multithreaded systems include the TinyOS TOSThread [25], Contiki multithreading [17] and mantisOS [12]. MantisOS is a purely traditional multithreaded WSN OS. TinyOS TOSThread and Contiki multithreading are the multithreaded systems which are developed complementarily on the basis of the event-driven kernels (Figure 15), and the purpose of these two systems is to add the preemption functionality (required by the long-time tasks which cannot be split by the split-phase programming [9]) into the fully event-driven systems.

**Figure 16.**
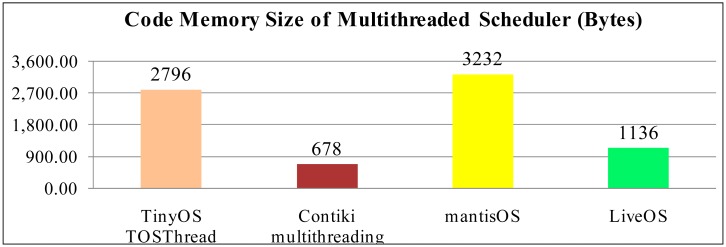
Code memory size of the multithreaded scheduler in different WSN OSes. Multithreaded scheduling system is complementally implemented in the event-driven system TinyOS and Contiki.

**Figure 17.**
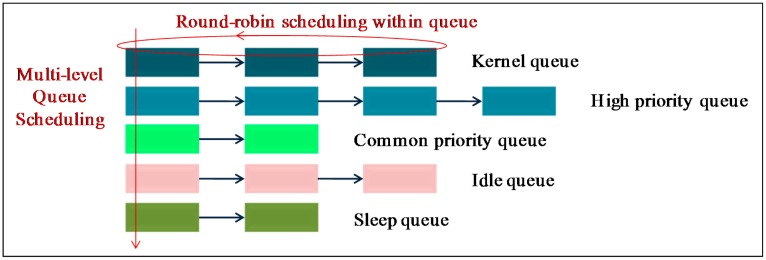
Multi-level round-robin scheduling in mantisOS. By using these scheduling queues, threads in mantis OS can be managed efficiently.

In Figure 16, the code memory size of the different systems is shown. MantisOS consumes the most code memory resources, this is because it is a pure multithreaded WSN OS. In mantisOS, all system tasks (either RT tasks or non-RT tasks) are executed by threads, thus the thread number can be large. In order to manage these threads efficiently, the multi-level queue scheduling approach is used (Figure 17). This approach improves the scheduling efficiency, but increases the code memory size. Code memory size of TinyOS TOSThread is also large because the application space is decoupled from the underlying system space in TOSThread [25] (Figure 15a), and the message communication mechanism is applied for the interaction between the application space and the system space. With these mechanisms, the application development process in TinyOS is simplified, but the software architecture becomes more complicated, and more code memory resources are needed. Contiki multithreading system is simply implemented as a library, and the code memory size is the minimum. In LiveOS, the dynamic-stack hybrid scheduling mechanism is implemented. The code memory size of LiveOS scheduler is not large if compared to the others, and there are two main reasons: First, the thread number in LiveOS is small (all non-RT tasks are executed by only one thread after the hybrid scheduling mechanism is applied, Figure 11), thus thread scheduling architecture is simple, and the code memory size of the multithreaded scheduler is not large (986 bytes). Second, the event control flag mechanism (Figure 11) rather than the scheduling queue mechanism (Figure 1) is used in LiveOS for the implementation of the event-driven scheduler. In LiveOS, every non-RT task has one corresponding event control flag. Once an event is triggered, the related control flag will be set. An event-driven scheduler polls the control flags in loop. If a control flag is observed to be set, the related task will be executed. By using this mechanism, the event-driven scheduling architecture becomes simple, and the event-driven scheduler costs only 150 bytes of code memory in LiveOS (TinyOS event-driven scheduler: 178 bytes; Contiki event-driven scheduler: 936 bytes).

#### 3.4.2. Data Memory Consumption of the Multithreaded Scheduling System

The data memory cost (RAM cost) of the multithreaded scheduler can be denoted as follows:

(3) $$E=S_{\text{DATA}/\text{BSS}}+S_{\text{TCB}}\times N_{\text{TCB}}+S_{\text{STK}}\times N_{\text{STK}}$$

in which *S_DATA_*_/_*_BSS_* represents the data memory size of the DATA/BSS sections, *S_TCB_*, *N_TCB_*, *S_STK_* and *N_STK_* represent respectively the structure size of thread control block (TCB), the number of reserved thread TCBs, the size of thread stack and the number of thread stacks.

In LiveOS, all non-RT tasks are performed by only one thread (Figure 11). Thus, *N_TCB_* and *N_STK_* are smaller if compared to those in the traditional multithreaded OSes. It can be assumed that *N_TCB_* and *N_STK_* are respectively 13 and 10 in TinyOS/Contiki/mantiOS, whereas 10 and 6 in the LiveOS.

Thread stacks in LiveOS are dynamically allocated on the requirement, rather than statically pre-reserved in terms of the worst case. Therefore, *S_STK_* in LiveOS is smaller if compared with that in traditional multithreaded OSes. It can be assumed that *S_STK_* is 128 bytes in TinyOS/Contiki/mantiOS (128 bytes is the size which is used by mantisOS on the 8-bit AVR microcontroller), whereas it is 86 bytes in LiveOS(86 bytes is calculated by averaging the stack sizes of five different threads in mantisOS: *led*_*example* thread, *radio*_*demo* thread, *testbed*_*send* thread, *testbed*_*sense* thread and *testbed*_* multihop*_*relay* thread).

In terms of the assumptions above, the data memory sizes of the different multithreaded OSes can be computed, and the results are shown in Table 1.

From the results in Table 1, it is shown that the stack memory cost of LiveOS has been reduced 59.7% if compared to that of traditional multithreaded OSes. This is because the LiveOS stack number is decreased by using the hybrid scheduling mechanism. Meanwhile, each LiveOS stack size is decreased by using the stack-shifting dynamic allocation mechanism.

#### 3.4.3. Scheduling Efficiency

The scheduling efficiency of the multithreaded scheduler can be evaluated by the execution clock cycles.

In a traditional multithreaded OS, the scheduling process includes the saving of common context data, the selection of the next thread to be executed, and the restoring of the next thread's common context data. In LiveOS, the dynamic-stack scheduling is used, and the scheduling process includes the saving of common context data, the selection of next thread to be executed, the shifting of next thread's stack, and the restoring of the next thread's common context data. Namely, the LiveOS dynamic-stack system has the extra scheduling overhead on performing the stack shifting (Table 2).

The clock cycle of saving and restoring the common context data in different WSN OSes is approximate. In TinyOS/TOSThread, Contiki multithreading, mantisOS and LiveOS, it is respectively 77, 93, 99 and 88.

The clock cycles of selecting the next thread depends on the scheduling algorithm. In TinyOS and Contiki, the round-robin algorithm is used, and the selecting clock cycles are 26 and 22 respectively. In mantisOS, the multi-level queue round-robin scheduling mechanism is implemented (Figure 17), and the selecting clock cycle is 72. In LiveOS, the RMS scheduling algorithm is applied, and the selecting clock cycle is (5 + 9*n*), in which *n* represents the searching steps of the next ready thread in the thread queue (Figure 11). Since the thread number in LiveOS is small, *n* can be assumed as 3. In this case, the clock cycle of selecting the next thread in LiveOS is 32.

The clock cycle of the stack shifting in LiveOS is (28 + 8 × *p*), in which *8* represents the clock cycle of copying one-byte memory, and *p* represents the size of the private context data. Memory defragmentation is needed in LiveOS in some cases, and the defragmentation clock cycle is (53 + 8 × *m* × (*p* + *c*)), in which *m* represents the number of the stacks that are coalesced during the defragmentation process, *c* represents the size of common context data (35 bytes on AVR ATmega1281 microcontroller [28]). In terms of the assumption in Table 1, *p* can be assigned as 51 (51 = 86 − 35). Since thread number is small in LiveOS (Figure 11), *m* can be assigned as 2. Consequently, the clock cycle of thread scheduling in LiveOS can be calculated:

- In case stack shifting is unnecessary (Figure 9), the scheduling clock cycle of LiveOS is 120 (120 = 88 + 5 + 9 *n*).
- In case stack shifting is needed, but the memory defragmentation is not performed, the scheduling clock cycle of LiveOS is 556.
- In case stack shifting is needed, and the defragmentation should also be performed before the shifting operation, the scheduling clock cycle of LiveOS is 1985.

In case ATmega1281 microcontroller works under the frequency of 16MHz, the time cost during the thread scheduling process in above three cases are respectively 7.5 μs, 34.75 μs, and 124.06 μs.

### 3.5. Discussion

Three different stack allocation approaches have been discussed in this section: the traditional stack allocation approach, stack-size analysis allocation approach (Section 2.1.1) and LiveOS stack-shifting dynamic allocation approach (Section 3.1).

Traditional stack allocation approach allocates the stacks by static pre-reservation, and the pre-reserved size is assigned heuristically. To avoid the memory overflow problem, the size of each stack needs to be assigned to a large value in terms of the worst case. Consequently, much stack memory can be wasted.

**Figure 18.**
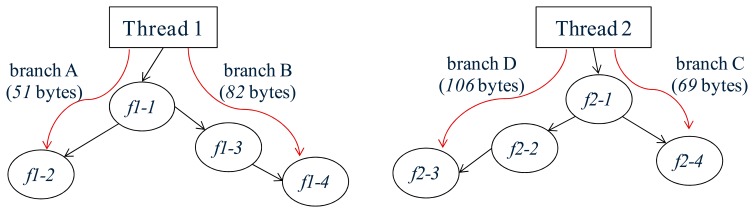
Thread1 has two execution branches: A and B. Stack sizes required by these two branches are 51 bytes and 82 bytes respectively. Thread2 also has two execution branches: C and D, and stack sizes required by these two branches are 69 bytes and 106 bytes, respectively.

Stack-size analysis approach targets to ease the stack memory waste by assigning each stack size to the actual value that is required (but not assigning the stack size heuristically). Compared to the traditional allocation approach, this approach is more optimized in the memory cost. However, the static scheme is still used and the memory waste problem still exists. For example, in Figure 18, thread1 has two execution branches (*A* and *B*). In case the run-time stack size required by these two branches are respectively 51 bytes and 82 bytes, then 82-byte (the largest stack size of all branches) stack memory will be allocated. This result remains the same even if branch *B* is executed rarely. In addition, the stack-size analysis approach cannot effectively work in several cases (Section 2.1.1); its availability is therefore limited in some WSN systems.

LiveOS stack-shifting dynamic allocation approach is different from the above two in that the stacks are allocated dynamically during the run-time on the requirement of the threads. Therefore, the memory waste problem existed in the above static allocation schemes can be avoided.

It is assumed that two threads in Figure 18 are executed on the 8-bit AVR microcontroller. If the traditional stack allocation approach is used, the stacks will take up 256 bytes of memory (128-byte stack size is used in mantisOS). If the stack-size analysis approach is used, the stacks will take up 188 bytes of memory (188 = 86 + 102, Table 3). However, if the LiveOS dynamic-stack approach is used, the stacks will be dynamically allocated, and the stack size changes between 120 bytes and 188 bytes (120 = 51 + 69, 188 = 82 + 106, Table 3). From this example, it can be shown that memory cost can be optimized efficiently in LiveOS by using the stack-shifting dynamic allocation approach.

Although the LiveOS dynamic-stack mechanism has higher scheduling overhead when compared to the traditional static-stack mechanism (Table 2), it is still feasible for use on the WSN nodes. This is because most WSN applications are not complicated and the real-time task number is not large. Since the real-time task number is small and the preemption is performed mainly among the real-time tasks in LiveOS (Figure 11), the scheduling overhead of LiveOS will not be significant, and this makes LiveOS suitable to be used on the resource-constrained WSN nodes.

Typically, there can exist some WSN applications in which the number of real-time threads is very large. In these applications, the scheduling overhead of LiveOS will be high, and LiveOS will not be efficient. This case is normal because the design purpose of LiveOS focuses on the optimization of the memory cost, rather than the improvement of the real-time scheduling performance.

## 4. LiveOS Energy Conservation Using the Multi-Core Hardware Technique

The LiveOS energy conservation approach is different from the other approaches in that the energy is conserved not only from the software aspect, but from both the software aspect and the multi-core hardware aspect. In this section, two kinds of LiveOS multi-core energy conservation mechanisms are presented. One is the multi-core context-aware conservation approach, presented in Section 4.1. The other is the multi-core “power off/wakeup” conservation approach, presented in Section 4.2. In Section 4.3, a discussion on the WSN multi-core technique is presented.

### 4.1. Multi-Core Context-Aware Energy Conservation Mechanism

The concept of LiveOS multi-core context-aware conservation mechanism derives from the experimental results that different microcontrollers are energy efficient in executing different tasks. Commonly, microcontrollers with powerful computation ability are more energy-efficient in executing complicated tasks while microcontrollers with weak computation ability are more energy-efficient in executing lightweight tasks. In Table 4, two microcontrollers (8-bit AVR ATmega1281 [29] and 32-bit ARM AT91SAM7Sx [30]) are selected for this energy cost evaluation. From the results, it is shown that different microcontrollers have different advantages in executing different tasks.

Based on the results above, a multi-core WSN node can be designed and implemented. On this node, several feature-different microcontrollers can be equipped. When a task needs to be executed, the microcontroller which is the most energy efficient to execute this task can be selected to enter working mode, whereas the other microcontrollers can be set to inactive mode (fall asleep or be powered off). By doing this, WSN nodes can become context aware to the tasks being executed, and the energy resources can be utilized more efficiently. It is assumed that *N* microcontrollers and *M* tasks exist on the multi-core node. In case these tasks are executed by the single-core node (equipped with only one microcontroller), the energy cost by these nodes will be:

(4) $$\begin{array}{l} \begin{array}{ll} {\text{E}}_{\text{node}-1}=\overset{M}{\underset{\text{i}=1}{\text{∑}}}{\text{E}}_{\text{controller}1-\text{task}i} & \cdots \cdots \end{array}\\ {\text{E}}_{\text{node}-N}=\overset{M}{\underset{\text{i}=1}{\text{∑}}}{\text{E}}_{\text{controller}N-\text{task}i} \end{array}$$

in which E_controller_*_N_*_-task_*_i_* represents the energy cost of executing the task *i* on the microcontroller *N*. However, if these tasks are executed by the multi-core node, the energy cost will be:

(5) $${\text{E}}_{\text{multi}-\text{core}}=\sum_{\text{i}=1}^{M}{\min(\text{E}}_{\text{controller1}-\text{task}i}{,\text{E}}_{\text{controller2}-\text{task}i},\ldots {,\text{E}}_{\text{controller}N-\text{task}i})$$

Since E_multi-core_ ≤ min(E_node-1_,E_node-2_,……,E_node-N_), multi-core node can be more energy efficient.

**Figure 19.**
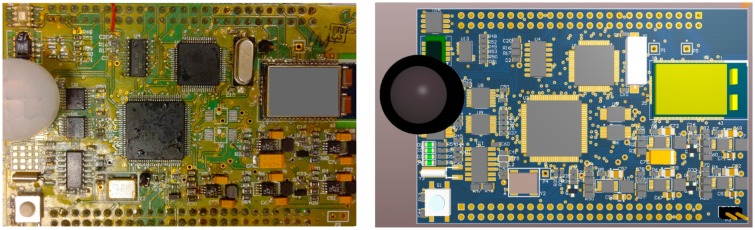
Prototype board of the multi-core node EMWSN.EMWSN is equipped with three microcontrollers: AVR ATmega1281, ARM AT91SAM7Sx and IGLOO nano FPGA.

**Figure 20.**
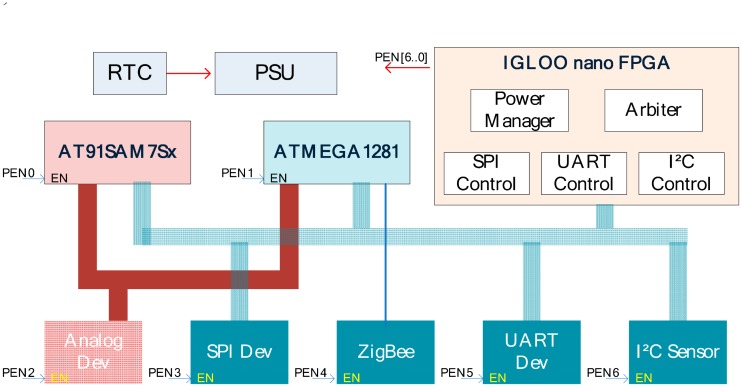
Circuit diagram of the multi-core EMWSN node. The working modes of different microcontrollers can be configured.

In terms of the concept above, a multi-core WSN node EMWSN is implemented (Figure 19). EMWSN is equipped with three microcontrollers: 8-bit AVR ATmega1281 [29], 32-bit ARM AT91SAM7Sx [30] and the low-power IGLOO nano FPGA [31] (Figure 20). ATmega1281 and AT91SAM7Sx function as the main WSN microcontrollers, and are in charge of performing most WSN tasks (sensing, data storage, signal processing, transmission, *etc.*). The IGLOO nano FPGA, which has ultra-low power (as low as 2 μW in Flash*Freeze mode [32]) and small footprint packages (as small as 3 × 3 mm), functions as the auxiliary microcontroller. With the configurable auxiliary IGLOO FPGA, the working modes (active mode or inactive mode) of the WSN microcontrollers (ATmega1281 and AT91SAM7Sx) can be adjusted without any wired change.

By using the multi-core architecture, EMWSN node becomes context aware to the WSN tasks. In case the sensing and wireless transmission tasks need to be performed, the AVR microcontroller is configured to enter the active mode (in terms of results in Table 4) and the ARM microcontroller is configured to enter the inactive mode. In the case that the signal processing task and the data storing tasks need to be performed, the AVR microcontroller will be set into inactive while the ARM microcontroller will be set active. By so doing, energy resources can be conserved in comparison to the single-core AVR node or ARM node.

To evaluate the performance of the multi-core context-aware conservation mechanism, energy costs of the multi-core EMWSN node, the single-core AVR node (equipped with ATmega1281), the single-core ARM node (equipped with AT91SAM7Sx) and the single-core TelosB node are compared. In these cases, the WSN tasks performed by these nodes are as follows: temperature and light sensors are sampled every 3 min (the same frequency as TelosB used in the article [33]), and the collected sensing data is stored first, before being processed and transmitted. If nodes are powered by a pair of AA Lithium/Iron Disulfide (Li/FeS2) 3000 mAh batteries, the lifetime of the single-core AVR node, single-core ARM node, single-core TelosB node and multi-core EMWSN node on executing these tasks are respectively 829 days, 382 days, 945 days and 1276 days. From this result, it is shown that energy cost of the multi-core EMWSN node can be reduced 35.03%, 70.06% and 25.94% respectively if compared to that of the single-core AVR node, ARM node and TelosB node.

### 4.2. Multi-Core “Power-Off/Wake-Up” Energy Conservation Mechanism

**Figure 21.**
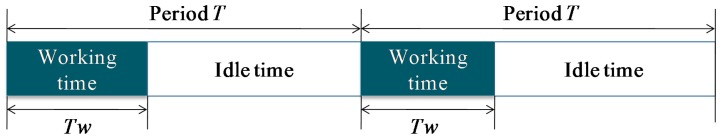
Node works intermittently. During the working time, the WSN tasks will be performed.

Besides the multi-core context-aware conservation mechanism, another mechanism to conserve the energy resource in LiveOS is the multi-core “power-off/wakeup” approach (rather than the “sleep/wakeup” approach that most WSN OSes use, Section 2). The “power-off/wakeup” mechanism is appropriate when the following assumption conditions are satisfied:

*Assumption condition 1:* Nodes work at intervals. Namely, the status of the node switches between the working mode and the idle mode (Figure 21).
*Assumption condition 2:* Tasks are independent from each other. Namely, the node can be powered off during the idle period. Once being powered on, the tasks can still be performed correctly.
*Assumption condition 3:* Standby current of the working microcontroller is not low. Specifically, standby costs non-trivial energy resources even after the microcontroller enters the idle mode.

The above conditions can be satisfied in many WSN applications, e.g., in the precision agriculture WSN application, the node equipped with the ATMEGA1281 microcontroller wakes up every 30 min to collect the soil temperature data. Once the data collection task is completed, the node falls asleep. In this application, the nodes work periodically (condition 1 is satisfied), and the temperature collection tasks are independent from each other (condition 2 is satisfied). Moreover, the standby current of the ATMEGA1281 microcontroller is not ultra-low (ATMEGA1281 is 500 μA in active mode and 130 μA in standby mode [29]). Thus, non-trivial energy resources will still be consumed during the idle period (condition 3 is satisfied). In these applications, the LiveOS multi-core “power-off/wakeup” energy conservation mechanism can be effective.

The concept of LiveOS multi-core “power-off/wakeup” energy conservation is to use an ultra-low-power auxiliary microcontroller to manage the high-power working microcontroller. With the assistance of the auxiliary microcontroller, the high-power working microcontroller can be powered off directly (rather than fall asleep) during the idle period. Consequently, energy can be optimized.

Currently, most WSN nodes are single-core architecture. The single-core node needs to fall asleep during the idle period (for energy conservation, “sleep/wakeup” mechanism, Section 2). Single-core node cannot power it off when it is idle, otherwise, it can no longer be powered on. Although less energy will be spent by enabling the nodes to fall asleep, much energy will still be consumed if the standby current is not small and the sleep period is long. Ideally, the working microcontroller can be powered off (but not fall asleep) when the node enters the idle status, and then be woken up when the next working period arrives (“power-off/wakeup” mechanism). In so doing, energy resources can be conserved. In LiveOS, this energy conservation concept is implemented, and it is achieved by means of the multi-core WSN technique.

It is assumed that two microcontrollers are equipped on the multi-core node: the typical working microcontroller *A* and the auxiliary microcontroller *B*. Nodes work intermittently. The periodical time is *T*, and the working time is *Tw* (Figure 21).

If the traditional single-core node (node which is equipped with only the working microcontroller) is used, the node needs to fall asleep when it is idle, and the energy cost within one period is:

(6) E=EwA+EsA=U×IwA×Tw+U×IsA×(T−Tw)

in which *EwA* and *IwA* represent respectively the energy cost and the current value when the node is in working status, *EsA* and *IsA* represent respectively the energy cost and the current value when the node is in idle status, and *U* represents the voltage value.

However, after the LiveOS multi-core WSN technique is used, two microcontrollers can be equipped on the node: the working microcontroller *A* plus the ultra-low-power auxiliary microcontroller *B*. The functionality of microcontroller *B* is to control the power supply to microcontroller *A*. With the auxiliary microcontroller *B*, *A* can be powered off directly when the node becomes idle, and then be powered on when the WSN tasks need to be performed. By doing this, the energy cost within one period will be:

(7) $$E^{\prime }=\text{EwA}+\text{EwB}+\text{EsB}=U\times \text{IwA}\times Tw+U\times \text{IwB}\times Tw+U\times \text{IsB}\times (T-Tw)$$

in which *EwA*, *IwA*, *EwB* and *IwB* represent respectively the energy cost and the current value when *A* and *B* are in the working status, *EsB* and *IsB* represent respectively the energy cost and the current value when *B* is in the idle status, and *U* represents the voltage value.

In terms of the Equations (6) and (7), the energy conserved within one period by using the multi-core WSN node will be:

(8) $$e=E-E^{\prime }=U\times T\times [(1-\frac{Tw}{T})(\text{IsA}-\text{IsB})-\frac{Tw}{T}\times \text{IwB}]$$

The larger the value *e* is, the more energy will be conserved. Since *IsA* is commonly a constant value depending on the characteristics of microcontroller *A*. Moreover, in terms of the assumption condition 3, *IsA* is not a small value. Therefore, the energy can be conserved if the following conditions are satisfied:

Conservation condition 1: The current values of the auxiliary microcontroller in the working status (*IwB*) and the sleep status (*IsB*) are low. The lower the *IsB* and *IwB*, the more the energy resource can be conserved.
Conservation condition 2: Duty cycle of the working time (that is, the *Tw*/*T*) is small. The smaller the duty cycle value, the more the energy resources can be conserved.

To satisfy the condition 1, an ultra-low-power auxiliary microcontroller can be selected. As for condition 2, it can be well satisfied in many WSN applications, e.g., in the precision agriculture application, the nodes sense the environmental temperature every 30 min (T is equal to 1800 s) and the sensing work can be completed within 8 s (Tw is equal to 8 s). In this case, the duty cycle of the working time (*Tw*/*T*) is 0.004.

Typically, the node performs the short-time task within the long-time period. In this case, the value of working time duty cycle (*Tw*/*T*) is approximately zero, and the energy conserved by using the multi-core node will be:

(9) $$e=E-E^{\prime }=U\times T\times [\text{IsA}-\text{IsB}].$$

Under this condition, energy conserved by the multi-core “power-off/wakeup” mechanism depends linearly on the differential value between *IsA* and *IsB*.

**Figure 22.**
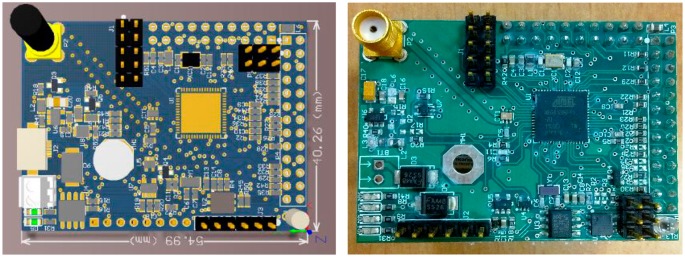
Prototype board of the multi-core node LiveWSN. Due to the ultra-low-power cost, tinyRisc is selected as the auxiliary microcontroller on the LiveWSN node.

Currently, this LiveOS “power-off/wakeup” conservation approach has been implemented on the LiveWSN node (Figure 22). LiveWSN node is equipped with the AVR ATmega1281 microcontroller (8-bit working microcontroller, 500 μA in active mode and 130 μA in standby mode. Namely, *IwA* is 500, *IsA* is 130) and the tinyRisc microcontroller (4-bit auxiliary microcontroller, 5.8 μA current in active mode and 3.3 μA current in standby mode: *IwB* is 5.8, *IsB* is 3.3), and the voltage of the node is 3V (*U* is 3). It is assumed that the node samples the temperature and light sensing data every 15 min (*T* is equal to 900 s), and the sensing work is completed within 10 s (*Tw* is equal to 10 s). Then, energy cost of LiveWSN within one period is 23.985 mW (23,985 = 3 × 500 × 10 + 3 × 5.8 × 10 + 3 × 3.3 × (900 − 10), Equation (7)). However, if the single-core node is used (only equipped with the AVR ATmega1281microcontroller), the energy cost within one period will be 362.1 mW (362,100 = 3 × 500 × 10 + 3 × 130 × (900 − 10), Equation (6)). From this result, it is shown that 93.38% of the energy resource can be conserved by the LiveWSN “power-off/wakeup” conservation approach in comparison to the single-core ATmega1281node.

### 4.3. Discussion

In this section, the mechanism of using the multi-core technique to achieve the energy conservation in LiveOS is presented. Besides the energy conservation, the multi-core technique can also be used to improve the OS real-time performance, the node reliability, *etc*.

The real-time performance can be improved in the multi-core node by distributing different tasks onto different microcontrollers to be executed concurrently. In the single-core node, if several real-time tasks become active simultaneously, these real-time tasks may not be schedulable even if an appropriate real-time scheduling algorithm is used (CPU computation resource is not enough). However, this problem can be solved on the multi-core node if these tasks are distributed.

In addition, the node reliability can be improved by using the multi-core WSN technique. If the single-core node is used, the node will fail once the microcontroller runs incorrectly. However, if the multi-core node is used, a high reliable auxiliary microcontroller can be used to manage the less reliable working microcontroller. Then, once the working microcontroller runs abnormally, the auxiliary microcontroller can catch this event and restart it. By doing this, the working microcontroller can be recovered from the faults and the WSN tasks can continuously be performed. Since the reliability of the auxiliary microcontroller is high, multi-core WSN node becomes more reliable if compared to the single-core node (auxiliary microcontroller can be high reliable as the program running on it is quite simple). Currently, this multi-core reliability approach has been applied to the LiveWSN node. With this approach, three deployed LiveWSN nodes have been working effectively for more than two years in the ISIMA garden.

Since many microcontrollers are equipped, as opposed to just one, the manufacturing price of the multi-core node will increase. However, the increase is not high. This is because most WSN microcontrollers are low-cost ones, e.g., the selling price of the AVR Atmega1281 microcontroller can be lower than five dollars (the selling price of some Ph sensors can be more than 100 dollars; the microcontroller is relatively cheaper). As a result, the price of the multi-core node is acceptable. More significantly, the integrated performance of the WSN node can be improved significantly by using the WSN multi-core technique (energy cost can be lowered, real-time performance and node reliability can be improved, *etc.*). Therefore, it is essential to use the multi-core technique in the WSN.

## 5. Conclusions

In this paper, the memory and energy optimization strategies for the multithreaded WSN OS LiveOS is presented. LiveOS uses the stack-shifting hybrid scheduling mechanism to achieve the memory optimization objective. By using the stack-shifting dynamic allocation, the size of each stack in LiveOS can be reduced. By using the hybrid scheduling, the number of the stacks in LiveOS can be decreased. As a result, LiveOS becomes memory-efficient and can cost the memory resource less than 50% of that in the traditional multithreaded mantisOS. With this memory optimization, LiveOS becomes feasible for running on the memory-constrained WSN nodes (TelosB, SenseNode, iLive, *etc.*). Not only optimized in terms of memory cost, LiveOS is also optimized for energy saving, and this is achieved by using the multi-core “context-aware” and multi-core “power-off/wakeup” energy conservation approaches. With these approaches, more than 30% of the energy resource can be conserved by LiveOS when compared to a typical single-core WSN system. With the energy optimization strategy, the lifetime of WSN nodes can be prolonged. Consequently, the WSN nodes become more competent to be deployed in harsh outdoor environments. Currently, an online demo about LiveOS can be accessed from the websites [34].

The ongoing work of LiveOS will focus on the following topics: (1) Optimizing the memory cost further by implementing a dynamic memory allocator which has high memory utilization efficiency, yet keeps low allocation overhead; (2) Combining the multi-core hardware technique and the state-machine validation software technique to improve the reliability of the WSN nodes; (3) Developing an integrated System on Chip (SoC) multi-core node. This node will be able to adapt to different WSN contexts by uploading the new software firmware. By using this integrated multi-core platform, the implementation of different nodes dedicated to different WSN applications can be avoided. Therefore, the node manufacture cost can be reduced, and the software development cycle can be minimized.

## Figures and Tables

**Table 1.** Data memory cost of different multithreaded WSN OSes.

**WSN OSes**	**RAM Cost Expression**	**Scenario**	**Stack Size (bytes)**	**Total RAM Cost (bytes)**
TinyOS TOSThread	22 + 16 × N_TCB_ + S_STK_ × N_STK_	NTCB = 13,	1280	1510
Contiki multithreading	8 + 8 × N_TCB_ + S_STK_ × N_STK_	NSTK = 10,		1392
mantisOS	40 + 22 × N_TCB_ + S_STK_ × N_STK_	SSTK = 128		1606

LiveOS	25 + 10 × N_TCB_ + S_STK_× N_STK_	NTCB = 10,	516	751
		NSTK = 6,		
		SSTK = 86		

**Table 2.** Scheduling clock cycles of different multithreaded WSN OSes.

**Thread Scheduling Operation**	**Execution Clock Cycles (ATmega1281, 16MHz)**			
	**TinyOS TOSThread**	**Contiki Multithreading**	**MantisOS**	**LiveOS**
Saving and restoring of *common* context data	77 + 26 = 103	93 + 22 = 115	99 + 72 = 171	88 + (5+9*n*)
Shifting of *private* context data	N/A			28 + 8 × *p*
Memory defragmentation	N/A			53 + 8 × *m* × (*p* + *c*)

**Table 3.** Stack memory size required by running the threads in Figure 18.

**Mechanisms**	**Stack Allocation**	**Size of Each Stack**	**Stack Size during Run-Time (bytes)**		
			**Thread1**	**Thread2**	**Total**
Traditional Multithreaded OS	Statically pre-reserved	Assigned heuristically (in terms of worst case)	128	128	256
Stack Size Analysis		Assigned by pre-calculation	82	106	188
Dynamic-stack LiveOS	Dynamically allocated	Assigned as required during run-time	51 when branch A, 82 when branch B	69 when branch A, 106 when branch B	between 120 and 188

**Table 4.** Energy consumed by executing different tasks on different microcontrollers.

**Tasks (Voltage: 3 V)**	**32-Bit ARM AT91SAM7Sx**			**8-Bit AVR ATmega1281**		
	
	**Current(mA)**	**Time(ms)**	**Energy(mJ)**	**Current(mA)**	**Time(ms)**	**Energy(mJ)**
Sensing task (Temperature/light sensing)	22.1	896	59.4	15.9	900	42.9

Data storage (100-byte Flash programming)	20.9	58	3.636	16.3	103	5.037

Sensing data processing (pure instruction execution)	19.7	5.0	0.296	9.9	268	7.96

Packet wireless transmission (ZigBee protocol)	21.8	139	9.091	20.9	132	8.276

Sleep	0.2	N/A	N/A	40 µA	N/A	N/A
